# Natural Selection of Human Embryos: Impaired Decidualization of Endometrium Disables Embryo-Maternal Interactions and Causes Recurrent Pregnancy Loss

**DOI:** 10.1371/journal.pone.0010287

**Published:** 2010-04-21

**Authors:** Madhuri Salker, Gijs Teklenburg, Mariam Molokhia, Stuart Lavery, Geoffrey Trew, Tepchongchit Aojanepong, Helen J. Mardon, Amali U. Lokugamage, Raj Rai, Christian Landles, Bernard A. J. Roelen, Siobhan Quenby, Ewart W. Kuijk, Annemieke Kavelaars, Cobi J. Heijnen, Lesley Regan, Nick S. Macklon, Jan J. Brosens

**Affiliations:** 1 Institute of Reproductive and Developmental Biology, Imperial College London, Hammersmith Hospital, London, United Kingdom; 2 Department of Reproductive Medicine and Gynecology, University Medical Center Utrecht, Utrecht, The Netherlands; 3 Department of Epidemiology and Population Health, London School of Hygiene & Tropical Medicine, London, United Kingdom; 4 Nuffield Department of Obstetrics and Gynecology, University of Oxford, Women's Centre, John Radcliffe Hospital, Oxford, United Kingdom; 5 Department of Obstetrics and Gynecology, the Whittington Hospital NHS Trust, London, United Kingdom; 6 Faculty of Veterinary Medicine, Utrecht University, Utrecht, The Netherlands; 7 Department of Reproductive and Developmental Health, Liverpool Women's Hospital, University of Liverpool, Liverpool, United Kingdom; 8 Laboratory of Psychoneuroimmunology, University Medical Center Utrecht, Utrecht, The Netherlands; 9 Division of Developmental Origins of Health and Disease, Princess Anne Hospital, University of Southampton, Southampton, United Kingdom; Indiana University, United States of America

## Abstract

**Background:**

Recurrent pregnancy loss (RPL), defined as 3 or more consecutive miscarriages, is widely attributed either to repeated chromosomal instability in the conceptus or to uterine factors that are poorly defined. We tested the hypothesis that abnormal cyclic differentiation of endometrial stromal cells (ESCs) into specialized decidual cells predisposes to RPL, based on the observation that this process may not only be indispensable for placenta formation in pregnancy but also for embryo recognition and selection at time of implantation.

**Methodology/Principal Findings:**

Analysis of mid-secretory endometrial biopsies demonstrated that RPL is associated with decreased expression of the decidual marker prolactin (PRL) but increased levels of prokineticin-1 (PROK1), a cytokine that promotes implantation. These *in vivo* findings were entirely recapitulated when ESCs were purified from patients with and without a history of RPL and decidualized in culture. In addition to attenuated PRL production and prolonged and enhanced PROK1 expression, RPL was further associated with a complete dysregulation of both markers upon treatment of ESC cultures with human chorionic gonadotropin, a glycoprotein hormone abundantly expressed by the implanting embryo. We postulated that impaired embryo recognition and selection would clinically be associated with increased fecundity, defined by short time-to-pregnancy (TTP) intervals. Woman-based analysis of the mean and mode TTP in a cohort of 560 RPL patients showed that 40% can be considered “superfertile”, defined by a mean TTP of 3 months or less.

**Conclusions:**

Impaired cyclic decidualization of the endometrium facilitates implantation yet predisposes to subsequent pregnancy failure by disabling natural embryo selection and by disrupting the maternal responses to embryonic signals. These findings suggest a novel pathological pathway that unifies maternal and embryonic causes of RPL.

## Introduction

Miscarriage is the most common complication of pregnancy. It is estimated that 30% of embryos are lost prior to implantation (pre-implantation loss) and a further 30% before 6 weeks gestation (pre-clinical/biochemical pregnancy loss) [1]. In addition, in excess of 10% of clinical pregnancies result in miscarriage, mostly prior to 12 weeks gestation, and 1–2% of couples experience recurrent pregnancy loss (RPL), defined as failure of 3 or more consecutive pregnancies [2]. Beside the physical trauma, miscarriage, and especially RPL, is associated with considerable psychological morbidity with a third of patients attending specialist clinics suffering from clinical depression [2]. Moreover, a history of RPL increases the risk of a variety of adverse obstetric outcomes in a subsequent ongoing pregnancy, including preterm delivery, premature preterm rupture of membranes, placenta praevia, low birth weight and congenital malformation [3].

Early pregnancy loss is widely viewed as a dichotomous disorder, attributed either to maternal factors or chromosomal errors in the conceptus. On the maternal side, numerous anatomical, endocrine, immunological, thrombophilic and genetic perturbations have been invoked to explain RPL, yet none are specific or prevalent [2], [3]. Moreover, for most of these conditions, the pathological mechanisms that account for persistent pregnancy wastage are entirely conjectural. Conversely, between 30 to 60% of miscarriages are attributed to fetal chromosomal anomalies [4]. These estimates are based on conventional karyotyping of fetal tissues, suggesting that the true incidence may be higher. However, the prevalence of gross mitotic chromosomal errors in preimplantation human embryos is also very high, affecting a staggering 90% of all embryos, even in young fertile women [5]. In other words, if chromosomal instability in the preimplantation embryo is the norm rather than the exception, then RPL could primarily reflect inadequate embryo selection, accounting for the high prevalence of aneuploidic miscarriages.

For most of the menstrual cycle, the endometrium is not primed for implantation. It only transiently acquires a receptive phenotype, starting approximately 6 days after the postovulatory progesterone surge and is estimated to last between 2 to 4 days [6], [7]. Arguably, a limited ‘implantation window’ synchronizes implantation with embryo development, which may serve as an important mechanism to select against developmentally impaired but potentially invasive embryos. Consistent with this concept, the population study of Wilcox *et al* [7] elegantly demonstrated that implantation beyond the normal period of endometrial receptivity is strongly associated with early pregnancy loss.

Although the luminal endometrial epithelium is the primary barrier in the implantation process, the progesterone responses in this cellular compartment that underpin the receptive phenotype are mediated by signals derived from the underlying stromal cells [8]. A striking feature of the human endometrium is that the acquisition of a receptive phenotype in the mid-secretory phase of the cycle coincides with decidualization of the stromal compartment, irrespective of pregnancy. Decidualization is characterized by transient local oedema, influx of macrophages and specialized uterine natural killer cells, angiogenesis, and the extraordinary transformation of resident endometrial stromal fibroblasts into secretory, epitheloid-like decidual cells [9]–[11]. From a functional perspective, the decidual process is indispensable for pregnancy in all species with invasive placentae as it establishes maternal immunologic tolerance to fetal antigens, protects the conceptus against environmental insults, and ensures tissue integrity and haemostasis during the process of trophoblast invasion and placenta formation [9], [10]. Further, decidualization of human endometrial stromal cells also bestows on the endometrium the ability to selectively recognize and respond to developmentally impaired embryos, as outlined in the accompanying paper by Teklenburg *et al*. in this journal.

Based on these observations, we hypothesized that impaired decidualization of the endometrium prior to conception predisposes for subsequent pregnancy failure, either by prolonging the implantation window, thereby disabling natural embryo selection, or by disrupting the maternal responses to embryonic signals. If correct, impaired decidualization and lack of natural embryo selection should not only lead to RPL but also be associated with paradoxical superfecundity, defined by persistent very short time-to-pregnancy (TTP) intervals.

## Results

### Endometrial Decidualization Is Impaired in RPL

**Figure 1:**
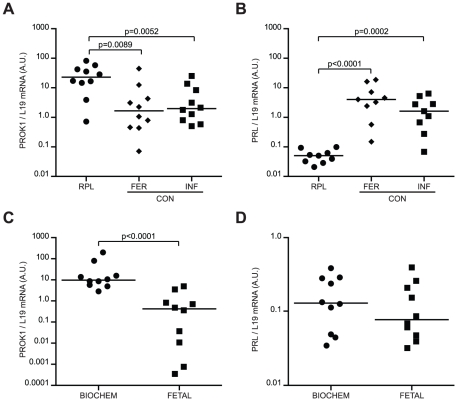
PROK1 and PRL transcript levels in timed endometrial samples of 10 RPL patients and 20 control (CON) subjects, consisting of 10 fertile (FERT) volunteers and 10 infertile (INF) patients without a history of RPL. PROK1 (*A*) and PRL (*B*) mRNA levels, normalized to L19 transcript levels, are expressed in arbitrary units (a.u.). Horizontal bars indicate the median expression in each group. PROK1 and PRL mRNA levels, respectively (*C & D*), in RPL patients with recurrent biochemical (biochem; n = 10) or fetal (n = 10) pregnancy failure. A ‘biochemical’ loss was defined as a miscarriage at 4–6 weeks gestation with ultrasound evidence of either an intrauterine pregnancy sac with no fetus or retained products of conception. A ‘fetal’ loss was defined as a pregnancy failure between 6–13 weeks gestation with prior ultrasound evidence of fetal development. Note the logarithmic y-axes.

We speculated that impaired decidualization of the stromal compartment may facilitate delayed implantation of compromised embryos by prolonging the window of endometrial receptivity, as suggested by the population study of Wilcox *et al* [7]. To test this hypothesis, we determined the expression levels of a recently identified key regulator of endometrial receptivity, prokineticin-1 (PROK1) [12], as well as prolactin (PRL), a classic decidual marker [11], [13]. Transcript levels were determined in endometrial biopsies, timed to span the implantation window, from RPL patients and controls, consisting of either fertile or infertile women without a history of recurrent pregnancy failure (Table S1). Compared with controls, RPL was associated with significantly higher endometrial PROK1 mRNA levels and approximately 100-fold lower PRL levels (Fig. 1A & B). Analysis of an independent sample set (Table S2) demonstrated that elevated PROK1 transcript levels are primarily associated with biochemical rather than fetal RPL, whereas no such association was found with PRL (Fig. 1C & D).

**Figure 2:**
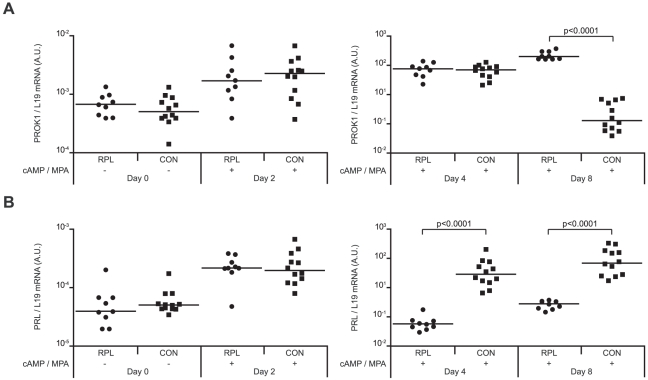
RPL is associated with aberrant expression of PROK1 and PRL in decidualizing primary ESC cultures. PROK1 (*A*) and PRL (*B*) mRNA levels, normalized to L19 transcript levels and expressed in arbitrary units (a.u.), were determined in undifferentiated ESCs (day 0) or cultures decidualized with 8-Br-cAMP and MPA (cAMP/MPA) for 2, 4 or 8 days. A total of 9 primary cultures were established from RPL patients and 12 from control (CON) subjects. Horizontal bars indicate the median expression in each group. Note the logarithmic y-axes.

To validate these *in vivo* observations, we established primary cultures from 9 RPL patients and 12 controls (Table S3). ESCs, purified from samples taken randomly in the cycle, were passaged once, allowed to grow to confluency, and then decidualized over a time-course lasting 8 days. PROK1 and PRL mRNA levels did not differ between the two groups in undifferentiated ESCs or in cells decidualized for 48 hours. However, after 4 days of differentiation, the rise in PRL transcript levels was several magnitudes higher in the control group when compared to RPL samples (Fig. 2). PROK1 levels continued to rise with comparable kinetics in all decidualizing cultures until day 8 when expression in the control but not RPL group declined markedly.

This expression profile in cultured cells supported the notion that an impaired decidual response, leading to prolonged endometrial receptivity and impaired embryo selection, is the primary uterine defect in RPL. Two additional observations are worth emphasizing. First, the magnitude of difference in PROK1 and PRL expression between the RPL and control group was not only high, at mRNA as well as at the secreted protein level (Fig. S1), but the pattern of expression in all samples corresponded to the clinical phenotype. Secondly, ESCs were purified from endometrial samples taken randomly in the cycle and maintained in prolonged culture, suggesting that the ability to mount a decidual response, perhaps more so than the signals responsible for differentiation, is perturbed in RPL.

### hCG Responses in Decidual Cells Are Disrupted in RPL

**Figure 3:**
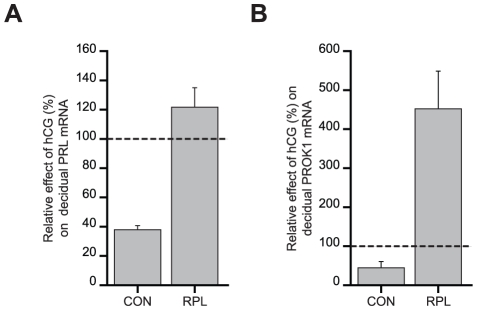
RPL is characterised by aberrant hCG responses in decidualizing ESCs. Primary cultures from RPL patients (n = 10) and control women (n = 10) were decidualized with 8-Br-cAMP and MPA for 72 hours in the presence or hCG or vehicle. The data show the percentage change (± SEM) in PRL (*A*) and PROK1 (*B*) mRNA levels upon hCG treatment of control and RPL cultures, relative to the expression levels in cultures treated with vehicle (dotted lines); *P*<0.005.

We speculated that aberrant differentiation of ESCs observed in RPL patients would interfere directly with the maternal response to embryonic signals. To test this hypothesis, we examined the effects of human chorionic gonadotropin (hCG), one of the earliest and most abundant glycoproteins secreted by embryonic trophoblast, on *PRL* and *PROK1* expresssion in 20 additional cultures (Table S4). ESCs were decidualized with 8-Br-cAMP and MPA for 72 hours in the presence or absence of hCG. As reported previously [14], hCG strongly inhibited PRL mRNA levels in all control cultures (Fig. 3A). In contrast, this response was without exception reversed in RPL samples and characterized by a modest but significant increase in PRL transcripts upon hCG treatment. PROK1 mRNA levels were also inhibited by approximately 50% upon hCG treatment of decidualizing control cultures whereas the opposite response, a 4-fold increase, was observed in RPL cultures (Fig. 3B). The data suggest that perturbed endometrial preparation prior to conception will have profound consequences on embryo-maternal interactions in pregnancy.

### RPL Is Associated with Short Time-To-Pregnancy

**Figure 4:**
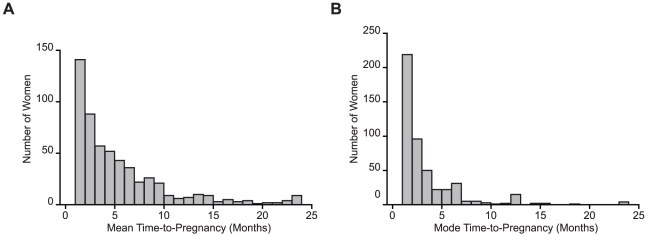
Woman-based analysis of time-to-pregnancy (TTP) in 560 women with a history of ≥3 consecutive first trimester miscarriages. Histogram representing woman-based analysis of the mean (*A*) and mode (*B*) TTP in patients with RPL.

RPL patients often report short periods between pregnancies, which is the predictable clinical correlate of impaired embryo recognition and selection. To substantiate this observation, we analysed the time-to-pregnancy (TTP), expressed in months, in 2076 pregnancies reported by 560 women with a history of 3 or more consecutive miscarriages. Woman-based analysis of the mean and mode TTP showed that many RPL patients are highly fecund (Fig. 4 and Table S5). Multivariate logistic regression analysis demonstrated that fecundity is further enhanced in the subgroup of patients with 5 or more recurrent miscarriages (odds ratio: 2.0; 95% confidence interval 1.1–3.4; *P* = 0.01 when adjusted for maternal age). In the absence of a suitable control group, we compared the observed incidence of achieving 3 or more pregnancies within 1, 3, or 6 months with the predicted likelihoods, based on an average MFR of 20% [15]. As shown in Table 1, the observed incidence of persistent short TTP intervals in RPL patients was much higher than predicted. In fact, 40% of RPL patients could be considered ‘superfertile’, defined by a mean TTP of less than 3 months [15]. Interestingly, maternal age was not an important confounding factor for TTP in RPL patients, which further suggests that implantation, rather than fertilization, is the major rate-limiting step in the fecundity of fertile couples.

**Table 1:** Proportion of women achieving ≥3 consecutive pregnancies within 1, 3, or 6 months.

	1 month	3 months	6 months
Predicted:	0.8%	8%	41%
RPL patients:	13%	41%	68%

Predicted likelihoods are based on a MFR of 20%; *P*<0.0001.

## Discussion

Our study provides compelling evidence that impaired decidual programming of the endometrium is an important mechanism underlying RPL. This is based primarily on the observation that expression of *PRL*, a highly sensitive and specific decidual marker gene in the endometrium [13], [16], is grossly impaired in RPL patients, both *in vivo* as well in primary cultures subjected to a decidualizing stimulus. However, we also obtained evidence that uterine receptivity is enhanced and prolonged in RPL patients, which in turn may facilitate delayed implantation of compromised embryos. Differentiating ESCs abundantly express PROK1, a cytokine that promotes embryo-uterine interaction via induction of leukaemia inhibitory factor (LIF) in endometrial epithelial cells [12]. The levels of this pro-implantation cytokine were significantly higher in timed endometrial samples from RPL patients, especially in women with a history of predominantly very early (biochemical) pregnancy losses. More strikingly, the induction of PROK1 in decidualizing endometrial cultures from control women was transient, with levels declining after 4 days of differentiation, whereas the levels continued to rise in cultures established from RPL patients. The concept of enhanced endometrial receptivity in RPL is supported by a previous study demonstrating that affected women express lower levels of mucin 1, an anti-adhesion molecule that contributes to the barrier function of luminal epithelium [4], [17].

We also found that RPL is further associated with paradoxical endometrial responses to embryonic hCG signalling, characterized by induction rather than inhibition of PRL and PROK1 expression in decidualizing ESCs. The ability of hCG to inhibit decidual PRL expression is in agreement with a previous study [14]. Interestingly, hCG has been shown to transiently induce PROK1 in Ishikawa cells, a cancer cell line widely used as model for uterine epithelial cells, which was followed by increased LIF expression [12]. Combined with our data, these observations indicate that under normal circumstances, hCG signalling may first transiently enhance uterine receptivity upon contact of the embryo with the luminal epithelium but upon implantation antagonize continuous receptivity by inhibiting decidual PROK1. The mechanism of normal hCG signaling in decidual cells remains to be defined [18], which is a prerequisite for understanding the disrupted transcriptional responses in RPL. Irrespective of the underlying pathway, a dysregulated response in pregnancy is likely to jeopardize the integrity of the feto-maternal interface as hCG, besides its luteotrophic role in early pregnancy, directly stimulates angiogenesis and limits cell death responses in the maternal decidua [19]–[21].

Although our expression analysis was confined to *PRL* and *PROK1*, the data demonstrate that endometrial programming in preparation of pregnancy is both quantitatively and qualitatively different in RPL, characterized by impaired decidualization of resident stromal cells, prolonged endometrial receptivity and a dysregulated maternal response to embryonic signals. This pathological pathway explains several established clinical features of RPL. For example, in view of the excess of chromosomally abnormal preimplantation human embryos [5], the likelihood of euploidic pregnancy failure can be predicted to increase with the number of miscarriages, which is indeed the case [22]. As mentioned, failure to limit the window of endometrial receptivity also accounts for delayed implantation of severely compromised embryos and subsequent very early pregnancy loss [7]. A primary defect in the decidual response may further explain why the incidence of miscarriage drops dramatically after 12 weeks gestation [2]. The last few weeks of the first trimester of pregnancy are particularly perilous for the conceptus as it coincides with intense vascular remodelling, the onset of placental perfusion, and a dramatic increase in oxygen tension at the feto-maternal interface [23]. Reactive oxygen species trigger a pro-apoptotic pathway in undifferentiated ESCs, which is selectively silenced upon differentiation into decidual cells [24]. Thus an adequate decidual response is critical to prevent cell death, necrosis, and bleeding at the feto-maternal interface, especially when challenged by oxidative stress signals towards the end of the first trimester. Finally, the concept of defective embryo selection explains why some RPL patients appear to be exceptionally fertile.

Biologic fertility is measured using TTP [25]. Based on an average MFR of 20%, a simple mathematical model predicts that 74%, 93%, and 100% of normally fertile couples will conceive in 6, 12, and 24 months, respectively [15]. Along the same lines, moderate and severe subfertility are defined by MFR of 5% and 1%, respectively. On the other side of the spectrum is superfertility, characterized by a MFR of 60% or more. In this context, the term ‘superfertility’ refers to enhanced efficacy in achieving pregnancy but not increased life-births. Superfertile couples achieve 94% and 100% of pregnancies within 3 and 6 months, respectively [15]. It has been estimated that 79% of the population is fertile, 18% subfertile or infertile, and 3% superfertile [15], [26]. Our retrospective analysis of TTP revealed that 40% of RPL patients report very short time to conception for each pregnancy. Thus, the prevalence of ‘superfertile’ couples in this population appears to be considerably higher than expected. Arguably, a majority of patients did not, or at least not consistently, report very short TTPs but this is not unexpected as the likelihood of conception is dependent upon many additional variables, including timing and frequency of coitus and the presence of coexisting disorders, such as suboptimal sperm quality or ovulatory, tubal and uterine defects. Hence, additional well-controlled, prospective studies are warranted to test our assumption that the superfertile end of the fecundity spectrum is as much a pathological condition as subfertility.

A striking observation is that the aberrant expression of *PRL* and *PROK1* in timed endometrial biopsies from RPL patients is recapitulated upon differentiation of purified ESCs maintained in prolonged cultures. Such cellular ‘memory’ suggests that the endometrial decidual response is subject to epigenetic programming in humans [9]. Interestingly, inflammatory signals are important epigenetic modifiers [27], which raises the possibility that the tissue trauma associated with pregnancy loss, or even menstrual events between pregnancies, may provide cues that dynamically modulate subsequent decidual responses in the endometrium [9]. If the epigenetic basis of RPL is correct than profiling of DNA or histone modifications in endometrial biopsies could be used to identify women at risk of adverse pregnancy outcome prior to conception and to monitor the effectiveness of medical interventions.

In summary, decidualization of uterine tissues is indispensable for placenta formation in pregnancy. We now provide evidence that failure to express an adequate decidual phenotype disables embryo recognition and selection upon implantation, which may lead to shorter TTP intervals but also predisposes to persistent pregnancy failure. Established risk factors for RPL, such as antiphospholipid antibodies and endocrine perturbations, may directly impact on uterine function, although our data suggest that the primary defect lies in the ability of ESCs to mount a decidual response. Together, these findings establish a novel pathological pathway that unifies maternal and embryonic causes of RPL.

## Materials and Methods

### Ethics statement and patient selection

The Research Ethics Committee of each participating centre approved the study: Hammersmith and Queen Charlotte's & Chelsea Research Ethics Committee (1997/5065) and the Liverpool (Adult) Research Ethics Committee (REC reference number: 05/Q1505/147). Written informed consent was obtained from all participating subjects. All patients were investigated according to the standard clinic protocols [28], but the outcome of these routine investigations was not taken in account in either the recruitment into this study or in the analysis of the data.

### Timed endometrial biopsies

Women were asked to use commercially available urine LH kits and to contact the research team at the time of their LH surge. All samples were obtained 6–10 days after the LH surge with a pipelle sampling device. Each biopsy was divided and one portion snap-frozen in liquid nitrogen for RNA analysis. The other portion was fixed in formalin for histological dating using standard criteria. The demographic details of the RPL and control groups are summarize in Tables S1 & S2.

### Analysis of the decidual response *in vitro*

Primary ESC cultures were established from endometrial biopsies, taken randomly in the cycle, as previous described [29]. The demographic details of RPL and control patients are summarized in Table S3. Primary cultures were passaged once, allowed to grow to confluency, and then decidualized with 8-Br-cAMP (0.5 mM) and MPA (10^−6^ M) for 2, 4 or 8 days. At each time-point, supernatants were collected and frozen and cells harvested for mRNA analysis. Additional cultures were established from control and RPL patients (Table S4) and decidualized with 8-Br-cAMP and MPA for 72 hours in the presence or absence of 10 nM hCG (Sigma, UK).

### PROK1 and PRL measurements

Total RNA was extracted from frozen tissue or cell cultures using Trizol Reagent (Invitrogen, Paisley, UK), treated with DNAseI (Ambion, Inc., Austin, TX), reversed transcribed, and the resulting cDNA subjected to real-time quantitative PCR analysis using an ABI Prism 7700 Sequence Detection System (Applied Biosystems, Foster City, CA) with the following gene-specific primer pairs: L19-sense (5′-GCG GAA GGG TAC AGC AAT-3′) and L19-antisense (5′-GCA GCC GGC GCA AA-3′); PRL-sense (5′-AAG CTG TAG AGA TTG AGG AGC AAA C-3′) and PRL-antisense (5′-TCA GGA TGA ACC TGG CTG ACT A-3′); PROK1-sense(5′-GTG CCA CCC CGG CAG-3′) and PROK1-antisense (5′-AGC AAG GAC AGG TGT GGT GC-3′). Secreted PROK1 and PRL levels were determined using ELISAs (R&D systems, Abingdon, UK).

### Time-to-pregnancy (TTP) analysis

Woman-based analysis of TTP was performed on a cohort of patients attending a tertiary service level Recurrent Miscarriage Clinic at Imperial College Healthcare NHS Trust. TTP for each pregnancy was recorded by medical staff on a standardised pro forma questionnaire upon referral to the clinic. TTP data of 856 women, encompassing 4018 pregnancies, were extracted from the clinical notes and subjected to analysis. After excluding incomplete data sets, the final analysis was based on 560 fertile women with a history of 3 or more miscarriages, which included 132 patients with 5 or more miscarriages. All analyses, carried out using STATA statistical software (Version 10), were adjusted for maternal age at time of pregnancy. Sensitivity analyses excluding women with other pregnancy complications (e.g. ectopic pregnancy, terminations and multiple pregnancies) showed similar effects. The unit of analysis was a pregnancy.

### Statistical analyses

Student's *t*-test and Mann-Whitney U test were used to determine statistical significance between two groups. For multiple comparisons, ANOVA test with Bonferroni correction was used. *P*<0.05 was considered significant. The association between the relevant pregnancy outcome and TTP was explored using logistic regression analysis, with effects on risk being estimated by odds ratios with 95% confidence intervals. Since women could have more than one pregnancy outcome in the analysis, a robust method based on the “sandwich estimate” [30] was used to compute standard errors, with Wald tests to ascertain statistical significance of parameters [31].

## Supporting Information

Figure S1 RPL is associated with impaired PROK1 and PRL secretion by decidualizing ESCs. Secreted PROK1 (A) and PRL (B) levels accumulated over 48 hours in the supernatants of confluent primary ESC cultures decidualization with 8-Br-cAMP and MPA for 8 days. Primary cultures were established from 10 RPL patients and 10 control (CON) subjects. Horizontal bars indicate the median expression in each group.
(0.61 MB EPS)

Table S1 Analysis of timed endometrial biopsies - patient characteristics. The data presented are mean ± standard deviation. * indicates P<0.001.
(0.03 MB DOC)

Table S2 Analysis of fetal versus biochemical RPL - patient characteristics. The data presented are mean ± standard deviation. * indicates P<0.05. A ‘fetal’ loss was defined as a pregnancy failure between 6–13 weeks gestation with prior ultrasound evidence of fetal development. A ‘biochemical’ loss was defined as a miscarriage at 4–6 weeks gestation with ultrasound evidence of either an intrauterine pregnancy sac with no fetus or retained products of conception.
(0.03 MB DOC)

Table S3 Time-course analysis - patient and culture characteristics. The data presented are mean ± standard deviation. LMP  =  last menstrual period. * indicates P<0.001.
(0.03 MB DOC)

Table S4 hCG analysis - patient characteristics. The data presented are mean ± standard deviation. LMP  =  last menstrual period. * indicates P<0.001.
(0.03 MB DOC)

Table S5 Analysis of time-to-pregnancy (TTP) in women with RPL. SD  =  standard deviation; NS  =  not significant.
(0.03 MB DOC)
