# Supplementary material for: Natural Selection of Human Embryos: Impaired Decidualization of Endometrium Disables Embryo-Maternal Interactions and Causes Recurrent Pregnancy Loss
Source: PLoS One. 2010 Apr 21;5(4):e10287. doi: 10.1371/journal.pone.0010287 (PMC2858209; doi:10.1371/journal.pone.0010287)
Supplement: Table S1 — Analysis of timed endometrial biopsies - patient characteristics. The data presented are mean ± standard deviation. * indicates P<0.001. (0.03 MB DOC) [file pone.0010287.s002.doc]

**Table S1.** Analysis of timed endometrial biopsies – patient characteristics

|  | **Control** | | **RPL** (n=10) |
| --- | --- | --- | --- |
|  | **Fertile** (n=10) | **Infertile** (n=10) |
| Age (years): | 34 ± 2.9 | 32.9 ± 4.1 | 35.1 ± 2.9 |
| Live births: | 2.5 ± 1.4* | 0.2 ± 0.4 | 0.6 ± 0.7 |
| Miscarriages: | 0.3 ± 0.5 | 0 | 7.4 ± 6.1* |
