# Supplementary material for: Natural Selection of Human Embryos: Impaired Decidualization of Endometrium Disables Embryo-Maternal Interactions and Causes Recurrent Pregnancy Loss
Source: PLoS One. 2010 Apr 21;5(4):e10287. doi: 10.1371/journal.pone.0010287 (PMC2858209; doi:10.1371/journal.pone.0010287)
Supplement: Table S2 — Analysis of fetal versus biochemical RPL - patient characteristics. The data presented are mean ± standard deviation. * indicates P<0.05. A ‘fetal’ loss was defined as a pregnancy failure between 6–13 weeks gestation with prior ultrasound evidence of fetal development. A ‘biochemical’ loss was defined as a miscarriage at 4–6 weeks gestation with ultrasound evidence of either an intrauterine pregnancy sac with no fetus or retained products of conception. (0.03 MB DOC) [file pone.0010287.s003.doc]

**Table S2.** Analysis of fetal versus biochemical RPL - patient characteristics

|  | **Fetal RPL** (n=10) | **Biochemical RPL** (n=10) |
| --- | --- | --- |
| Age (years): | 36.6 ± 3.4 | 30.4 ± 7.4* |
| Live births: | 0 | 0 |
| Miscarriages: | 4.4 ± 1.2 | 4.0 ± 1.0 |
