# Supplementary material for: Natural Selection of Human Embryos: Impaired Decidualization of Endometrium Disables Embryo-Maternal Interactions and Causes Recurrent Pregnancy Loss
Source: PLoS One. 2010 Apr 21;5(4):e10287. doi: 10.1371/journal.pone.0010287 (PMC2858209; doi:10.1371/journal.pone.0010287)
Supplement: Table S3 — Time-course analysis - patient and culture characteristics. The data presented are mean ± standard deviation. LMP = last menstrual period. * indicates P<0.001. (0.03 MB DOC) [file pone.0010287.s004.doc]

**Table S3.** Time-course analysis - patient and culture characteristics

|  | **Control** (n=12) | **RPL** (n=9) |
| --- | --- | --- |
| Age (years): | 33 ± 4.9 | 34.1 ± 3.6 |
| Live births: | 0.4 ± 1 | 0.4 ± 0.7 |
| Miscarriages: | 0.1 ± 0.3* | 3.9 ± 1.2 |
| Day of biopsy from LMP: | 17 ± 5.6 | 16.2 ± 7.9 |
| Days in culture | 16 ± 5.1 | 16.3 ± 4.1 |
