# Supplementary material for: Natural Selection of Human Embryos: Impaired Decidualization of Endometrium Disables Embryo-Maternal Interactions and Causes Recurrent Pregnancy Loss
Source: PLoS One. 2010 Apr 21;5(4):e10287. doi: 10.1371/journal.pone.0010287 (PMC2858209; doi:10.1371/journal.pone.0010287)
Supplement: Table S4 — hCG analysis - patient characteristics. The data presented are mean ± standard deviation. LMP = last menstrual period. * indicates P<0.001. (0.03 MB DOC) [file pone.0010287.s005.doc]

**Table S4.** hCG analysis - patient characteristics

|  | **Control** (n=10) | **RPL** (n=10) |
| --- | --- | --- |
| Age (years): | 34.1 ± 3.1 | 32.3 ± 6.6 |
| Live births: | 0.4 ± 0.9 | 0.2 ± 0.4 |
| Miscarriages: | 0 | 7 ± 3.7* |
| Day of biopsy from LMP: | 17.6 ± 7.1 | 20.8 ± 5.3 |
