# Supplementary material for: Natural Selection of Human Embryos: Impaired Decidualization of Endometrium Disables Embryo-Maternal Interactions and Causes Recurrent Pregnancy Loss
Source: PLoS One. 2010 Apr 21;5(4):e10287. doi: 10.1371/journal.pone.0010287 (PMC2858209; doi:10.1371/journal.pone.0010287)
Supplement: Table S5 — Analysis of time-to-pregnancy (TTP) in women with RPL. SD = standard deviation; NS = not significant. (0.03 MB DOC) [file pone.0010287.s006.doc]

**Table S5.** Analysis of time-to-pregnancy (TTP) in women with RPL

|  | **≥3 miscarriages**  (n=560) | **≥5 miscarriages**  (n=132) | ***P*** |
| --- | --- | --- | --- |
| Number of pregnancies: mean ± SD | 5.0 ± 2.1 | 7.4 ± 2.7 | <0.0001 |
| Mean age group (years) | 30-34.9 | 30-34.9 | NS |
| TTP: mean ± SD (months) | 5.4 ± 4.9 | 4.5 ± 4.2 | <0.05 |
| TTP : mode ± SD (months) | 3.0 ± 3.4 | 2.2 ± 2.3 | =0.01 |
| TTP: median ± SD (months) | 4.6 ± 4.6 | 3.8 ± 3.9 | NS |
| Women with mean TTP ≤ 1 month (%) | 70 (13%) | 23 (17%) | NS |
| Women with mean TTP ≤ 1.5 months (%) | 104 (19%) | 30 (23%) | NS |
| Women with mean TTP ≤ 3 months (%) | 229 (41%) | 66 (50%) | NS |
| Women with mean TTP ≤ 6 months (%) | 381 (68%) | 97 (73%) | NS |
